# Effects of nonsurgical periodontal treatment on glycated haemoglobin on type 2 diabetes patients (PARODIA 1 study): a randomized controlled trial in a sub-Saharan Africa population

**DOI:** 10.1186/s12903-018-0479-5

**Published:** 2018-02-26

**Authors:** Nadia-Flore Tsobgny-Tsague, Eric Lontchi-Yimagou, Arnel Redon Nana Nana, Aurel T. Tankeu, Jean Claude Katte, Mesmin Y. Dehayem, Charles Messanga Bengondo, Eugene Sobngwi

**Affiliations:** 1 Department of periodontology, oral and maxillofacial surgery, Faculty of Medicine and Biomedical Sciences of The University of Yaoundé 1, Yaoundé, Cameroon; 2 National Obesity Center, Yaoundé Central Hospital, Yaoundé, Cameroon; 3 Diabetes Research and Training Center, Division of Endocrinology, Department of Medicine, Albert Einstein College of Medicine, Bronx, NY USA; 4 Laboratory of Molecular Medicine and Metabolism, Biotechnology Center, University of Yaoundé 1, Yaoundé, Cameroon; 5 Department of Internal Medicine and specialties, Faculty of Medicine and Biomedical Sciences of The University of Yaoundé 1, Yaoundé, Cameroon; 6 Department of Public Health, Faculty of Medicine and Biomedical Sciences of The University of Yaoundé 1, Yaoundé, Cameroon; 7 Dental surgery Unit, Yaoundé Teaching Hospital, Yaoundé, Cameroon

**Keywords:** Periodontitis, Diabetes, Non-surgical periodontal treatment, HbA1c, Inflammation, Glycemic control

## Abstract

**Background:**

There is a burglar association between diabetes and periodontitis. Many studies has shown that periodontitis treatment can help improving glycemic control in diabetes patients but little evidence of non-surgical treatment benefit is available in sub Saharan african diabetes patients. We aimed to assess the effects of non-surgical periodontal treatment (NSPT) of chronic periodontitis on glycaemic control in poorly controlled type 2 diabetes patients (T2D) in a sub-Saharan Africa urban setting.

**Methods:**

A total of 34 poorly controlled T2D patients with chronic periodontitis aged 51.4 ± 8.8 years (mean ± SD), with known duration of diabetes of 55.5 ± 42.6 months, and HbA1c of 9.3 ± 1.3% were randomly assigned to two groups. The treatment group (Group 1, *n* = 17) received immediate ultrasonic scaling, scaling and root planning along with subgingival 10% povidone iodine irrigation, whereas the control group (Group 2, *n* = 17) was assigned to receive delayed periodontal treatment 3 months later. Pharmacological treatment was unchanged and all participants received the same standardized education session on diabetes management and dental hygiene. The primary outcome was the 3-month change in HbA1c from baseline. Plaque index (PI), gingival bleeding index (GBI), pocket depth (PD), clinical attachment loss (CAL) were also assessed prior to, at 6 and 12 weeks after enrolment.

**Results:**

Two subjects in each group were excluded from the study. Data were analyzed on thirty patients (15 per group). Non-surgical periodontal treatment with education for better dental hygiene (group 1) significantly improved all periodontal parameters whereas education only (group 2) improved only the plaque index among all periodontal parameters. Immediate non-surgical periodontal treatment induced a reduction of HbA1c levels by 3.0 ± 2.4 points from 9.7 ± 1.6% at baseline to 6.7 ± 2.0% 3 months after NSPT, (*p* ˂ 0.001) but the change was not significant in group 2, from mean 8.9 ± 0.9% at baseline vs 8.1 ± 2.6% after 3 months (*p* = 0.24).

**Conclusion:**

Non-surgical periodontal treatment markedly improved glycaemic control with an attributable reduction of 2.2 points of HbA1c in poorly controlled T2D patients in a sub Saharan setting.

**Trial registration:**

ClinicalTrials.gov Identifier: NCT02745015 Date of registration: July 17, 2016 ‘Retrospectively registered’.

## Background

African and particularly sub-Saharan Africa is faced with a significant and increasing burden of type 2 diabetes (T2D) with high prevalence in urban population, in addition of having the largest proportion of undiagnosed patients in the world. This makes it one of the most vulnerable regions for this condition worldwide now and in coming decades [1–3]. The situation is worsened by the fact that majority of patients with diabetes in sub-Saharan Africa are not well controlled with less than 30% reaching recommended glycaemic targets [4]. Poor glycaemic control favours incidence and severity of acute and chronic infections such as oral microorganism attacks. Periodontitis is the commonest oral disease affecting population worldwide with greater prevalence, severity and extent in diabetes patients [5–8]. In the other hand, severe periodontitis may exacerbate diabetes-induced hyperglycaemia [9–12] suggesting a bidirectional relationship between periodontal disease and diabetes in relation with inflammation and insulin resistance [13–18]. Despite growing evidence on the benefit of periodontal treatment on glycaemic control in people living with diabetes [19–25], randomized controlled trial-derived evidence is lacking from Africa, the continent with the largest burden of infectious diseases and uncontrolled diabetes. Therefore, we carried out this study to assess the effects of non-surgical periodontal treatment on glycated haemoglobin in a sub-Saharan Africa type 2 diabetes populations.

## Methods

### Ethical considerations

This study was performed in accordance with the guidelines of the Helsinki Declaration and was approved by the Institutional Research Ethical Committee of the Faculty of Medicine and Biomedical Sciences of Yaoundé and by the institutional review board of the Yaoundé Central Hospital of Cameroon. All participants provided written informed consent.

### Protocol and registration

This project was registered on Clinical Trials.gov. Identifier N°: NCT02745015.

### Setting and study population

This single blinded randomized controlled trial was carried out from December 2014 to May 2015 at the National Obesity Center of the Yaoundé Central Hospital, a tertiary center for diabetes care in the capital city of Cameroon. A total of 34 poorly controlled T2D patients with moderate to severe chronic periodontitis according to the 2012 CDC-AAP classification, and having at least eleven teeth (> 11 teeth) in the mouth were included [26]. The participants were excluded if they had received periodontal treatment (scaling and root planning) or experimented any alteration of the diabetes treatment six months prior to the study. Exclusion criteria also included: onset of systemic diseases or an acute condition, use of immunosuppressive medications or others drugs or presence of conditions able to alter periodontitis clinical features (pregnant women, alcohol users, smokers and acute anemia).

### Randomization and blinding

The randomization was made using a permuted block method with a block size of six. This method consisted of drawing one block out of the six non-distinguishable blocks contained in a non-transparent bag without replacement. The blocks are divided into two equal types and marked of two letters A and B (A = treatment and B = control). Therefore the bag contained 3 blocks A and 3 blocks B. Participants were assigned to one group or the other depending on the block drawn by the researchers; who were aware of the block drawn. Our study population was therefore divided into two groups, the treatment group (*n* = 17 received periodontal treatment) and the control group (*n* = 17 received delayed periodontal treatment). The periodontal examiners were masked to participants’ assignment.

### Data collection

Clinical and periodontal examination was carried out at the first day before nonsurgical periodontal treatment and during the follow-up. After taking informations related to diabetes history and management or lifestyle habits; a clinical examination was done including weight, height and blood pressure measurement. Periodontal examination consisted on periodontal parameters estimation at baseline and during follow-up six and twelve weeks later. Blood samples were collected for biological assessment of glycated haemoglobin at baseline and 3 months later.

#### Anthropometric measurements

For all participants, we measured height to the nearest 0.5 cm, and weight in light clothes to the nearest 0.1 kg, and calculated body mass index (BMI) as weight in kg/height^2^ in m^2^. Blood pressure was measured with a validated automated blood pressure measuring device (OMRON HEM-757).

#### Periodontal examinations

All recruited patients underwent full periodontal assessment at baseline, 6 weeks after assigned treatment and 3 months after assigned treatment. Periodontal state was estimated using a periodontal chart made of four periodontal parameters namely O’Leary plaque index (PI), Ainamo and Bay gingival bleeding index (GBI), probing pocket depth (PD), and finally clinical attachment loss (CAL). O’Leary’s plaque index was used to evaluate the quantity of plaque and the Ainamo and Bay gingival bleeding index was used in the evaluation of gingival inflammation [27, 28]. PI and GBI measurements were performed at four sites (mesial, distal, buccal, and lingual) around each tooth except the third molars. PD and CAL measurements were performed at six sites around each tooth except the third molars using a PCP probe (PCP UNC 12, Hu-Friedy Manufacturing Co., Inc., Chicago, USA). PD was measured as the distance from the free gingival margin to the base of the periodontal pocket. CAL was measured as the distance from the cemento-enamel junction (CEJ) to the base of the sulcus or periodontal pocket [CAL = PD - (CEJ – gingival margin (GM)]. Where the GM was subject to recession and the CEJ was exposed, the distance from the CEJ to the GM was given a negative value. Where the GM covered the CEJ, the distance between the GM and CEJ was given a positive value [29].

#### Biochemical measurements

HbA1c was determined at baseline, prior to treatment and 3 months after assigned treatment through a random blood sample using an automated device (LabonaCheck™A1c HbA1c, CERAGEM MEDISYS INC, South Korea).

### Periodontal treatment

The nonsurgical treatment was a full mouth debridement performed in a single visit consisting of scaling, root scaling and planning. This mechanical therapy was done using a magneto-restrictive ultrasonic device (Denjoy Dus-1A®, Changsha, China) and Gracey’s curettes followed by a sub-gingival irrigation with a 10% povidone iodine solution. Additionally, all the subjects of the treatment group received dental floss and chlorhexidine gluconate 0.2% as mouth wash (10 ml twice daily for 5 days). All subjects were instructed in oral hygiene methods: using of the modified Bass technique for tooth brushing, and using of soft bristled toothbrush. Dental floss is a known additional measure for plaque control, unfortunately, in limited-resources settings like ours, people are unaware of the importance of this device. Therefore, it is not systematically used by the patients. To improve plaque control of patients in our context, dental floss was given in order to help them familiarize with the device and ensure that they use it properly. At recall visit, 6 months after enrolment all the participants were re-motivated and a professional prophylaxis including scaling and polishing was performed only for the subjects in treatment group.

No interventional treatment was given to the control group during the follow up, apart from the oral hygiene instructions. However at the end of the study all the participants in the control group received the treatment as the treatment group.

### Sample size calculation

The sample size calculation determined that 14 subjects per treatment arm would provide 90% power to detect a minimum difference of 1% (SD 0.8) change in HbA1c level between the treatment and the control group. To compensate for possible drop out during the study period, a sample of 17 subjects per arm (34 in total) was recruited.

### Statistical analysis

Data were analyzed using IBM SPSS Statistics for Windows, version 20.0. (Armonk, NY: IBM Corp.). Results were presented as counts with percentage and means ± SD. The chi-squared test was used to test association between qualitative variables. The non-parametric Mann-Whitney U-test was used to compare data between non-normally distributed variables and the parametric paired t-test to compare data between normally distributed variables respectively between study groups. A two-tailed *p* value ˂ 0.05 was considered statistically significant test was used to compare the changes between the treatment and the control groups.

## Results

**Fig. 1:**
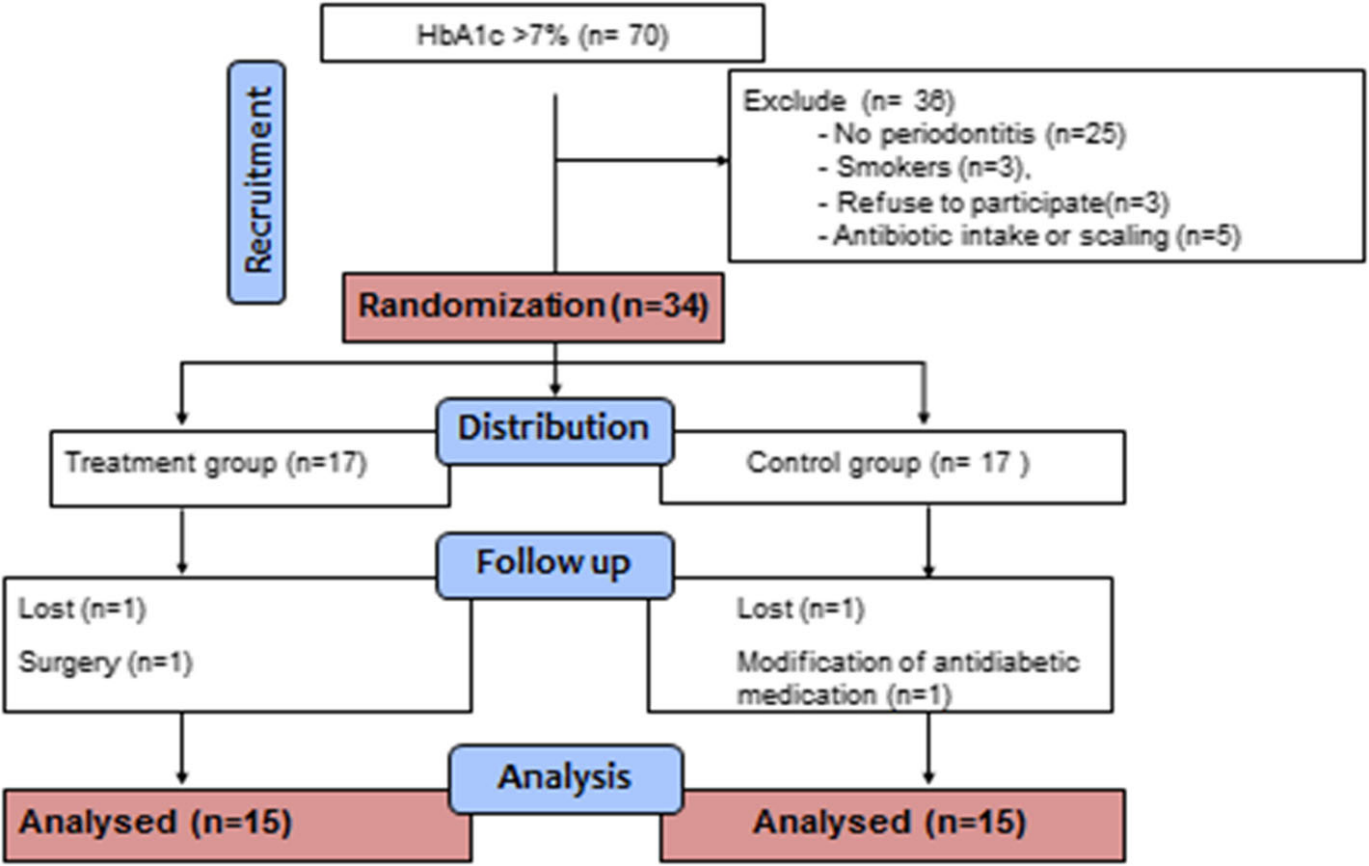
Diagram showing the flow of participants through each stage of the trial

We recruited 34 participants from a sample of 70 poorly controlled type 2 diabetes patients. Twenty five were ineligible because of the absence of periodontitis, three of them refused to participate, three were smokers, and five had a history of antibiotics intake or scaling less than three months ago. This left 34 eligible for randomization, as they gave their consent they were all randomized. Four participants were excluded after randomization (Fig. 1). There was no difference in the number of exclusion by study group (two in the treatment group and two in the control group).

### Characteristics of the study population at admission

As represented in Table 1, a total of 30 participants was analyzed in this study. Fifteen in periodontal treatment group (Group 1, *n* = 15) and fifteen in control group with delayed periodontal treatment (Group 2, *n* = 15). There was a good compliance in periodontal treatment group. At inclusion, the two groups were similar concerning diabetes history: duration, medications and complications, periodontal status, and HbA1c levels. (Table1).

**Table 1:** Comparison of two groups at baseline

	Treatment group	Control group	*p*-value
Sex** (M/F)	8/7	5/10	0.231
Age* (years)	51.2 ± 7.8	51.7 ± 9.9	0.677
Diabetes duration* (months)	60.0 ± 46.3	51.1 ± 39.6	0.618
BMI* (kg/m^2^)	29.2 ± 5.7	27.3 ± 5	0.580
PI* (%)	80.5 ± 13.1	79.3 ± 19.3	0.395
GBI* (%)	39.5 ± 18.9	37.2 ± 17.4	0.787
PD* (mm)	3.0 ± 0.4	3.1 ± 0.6	0.344
% sites˂4 mm	68.7	71.7	
% sites 4-6 mm	31.0	27.3	
% sites ˃6 mm	0.3	0.9	
CAL* (mm)	3.4 ± 0.5	3.3 ± 0.6	0.575
% sites ˂4 mm	57.0	64.4	
% sites 4-6 mm	41.7	33.6	
% sites ˃6 mm	1.3	2.0	
HbA1c* (%)	9.7 ± 1.6	8.9 ± 0.9	0.394
Methods used to control hyperglycemia**			
Diet	15	15	
OAD	13	7	0.025
Insulin	10	11	0.500
OAD + insulin	8	3	0.308
Complications**			
Neuropathy	6	6	0.645
Néphropathy	1	1	0.759
Rétinopathy	2	0	0.500
Diabetic foot	0	1	0.241

Data are mean ± standard deviation, *BMI* = Body mass index, *PI* = Plaque index, *GBI* = Gingival bleeding index, *PD* = Pockets depth, *CAL* = Cervical attachment loss, *OAD* = Oral antidiabetics

*: Mann- Whitney U test

**: *t*-test

### Glycated haemoglobin levels before and 03 months after non-surgical periodontal treatment

**Fig. 2:**
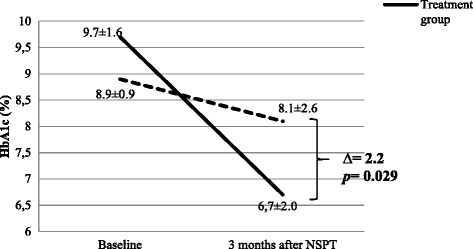
Changes in glycated haemoglobin during the follow up and between the two groups

After 3 months, we observed a significant improvement of glycaemic control in group 1 with a reduction of HbA1c levels of 3.0 ± 2.4 points [mean 9.7 ± 1.6% before vs 6.7 ± 2.0% after the NSPT, *p* ˂ 0.001] but no significant change in group 2 [mean 8.9 ± 0.9% before vs 8.1 ± 2.6% after the treatment, *p* = 0.238]. Finally, there was a relative reduction of 2.2 ± 2.5 points in HbA1c attributable to non-surgical periodontal treatment [*p* = 0.02] (Fig. 2).

### Periodontal parameters before and 03 months after non-surgical periodontal treatment

Treatment provides improvement in periodontal status in the treatment group with significant variation of all estimates used. Thus, plaque index dropped from 80.5% to 18.1% while gingival bleeding index was reduced by 34.1% from an average of 39.5% to 4.2% [*p* < 0.0001]. In the same way, average pockets depth decreased from 3 mm to 1.9 mm in this group for a 1.3 mm reduction in clinical attachment loss [*p* < 0.0001]. In contrast, the control group shows no significant variation in periodontal index status except plaque index which undergone a 15.6% significant reduction [*p* = 0.005] (Table 2).

**Table 2:** Periodontal parameters in each group before and 03 months after NSPT

Periodontal parameters	Before NSPT	3 months after NSPT	Δ	*p*-value
Treatment group				
PI (%)	80.5 ± 11.5	18.1 ± 15.1	56.5 ± 20.5	0.000
GBI(%)	39.5 ± 18.9	4.2 ± 4.7	34.1 ± 15.7	0.000
PD(mm)	3.0 ± 0.4	1.9 ± 0.3	1.1 ± 0.4	0.000
CAL (mm)	3.4 ± 0.5	2.1 ± 0.3	1.3 ± 0.5	0.000
Control group	At baseline	3 months of follow-up	Δ	*p*-value
PI (%)	79.3 ± 19.3	63.7 ± 15.3	15.6 ± 18.4	0.005
GBI(%)	37.2 ± 17.4	33.8 ± 15.9	3.3 ± 13.5	0.354
PD(mm)	3.1 ± 0.6	3.1 ± 0.6	0.0 ± 0.5	0.334
CAL (mm)	3.3 ± 0.6	3.3 ± 0.7	0.0 ± 0.5	1.0

Data are mean ± standard deviation; *NSPT*: non-surgical periodontal treatment, *PI* = plaque index, *GBI* = Gingival bleeding index, *PD* = pockets depth, *CAL* = Cervical attachment loss

### Compliance with medication

As the therapy included twice daily use of chlorhexidine mouth rinse for 5 days, one participant of the treatment group reported daily use, another one use for only three days, the 13 others reported a proper use.

### Adverse events

One case of tongue irrigation following chlorhexidine mouth rinse was reported in the treatment group.

## Discussion

After a twelve weeks follow-up of non-surgical treatment of chronic periodontitis by full-mouth ultrasonic scaling and root planning associated with sub gingival irrigation with 10% povidone iodine solution, we obtained a significant improvement of glycaemic control translated by a reduction of HbA1c levels to 30.9% of the baseline. The mean changes in HbA1c were found to be significantly different between the two groups. This permitted to conclude about the effect attributable to periodontal treatment which is a reduction of HbA1c of 2.2 points. Improvement of periodontal health characterised by an attachment gain of 1.3 mm with the reduction of plaque index and gingival bleeding index participates in reducing inflammation and consequently peripheral insulin resistance, as well as glycaemic level and glycated haemoglobin.

These results support those of previous studies which showed that elimination of periodontal infection can improve glycaemic control [19, 20, 30]. Loos 2005 [16] suggested that periodontal infection determine a low grade inflammation characterised by an up-regulation of pro-inflammatory cytokines like tumour necrosis factor-α (TNFα), interleukin-1 (IL-1) and IL-6 which is responsible of insulin resistance and poor glycaemic control [13, 14]. This means that periodontal treatment can improve glycaemic control in poorly controlled diabetes patients through the reduction of pro-inflammatory cytokines blood levels. These evidences come from many authors including Sun et al. [24], who showed that surgical and non-surgical periodontal therapy combined with systemic antibiotics intake improved glycaemic control, reduced serum inflammatory cytokines levels and insulin resistance in moderately poorly controlled T2D patients [24, 31–33].

However the mean change in HbA1c seems to be larger than the one found in similar studies in diabetic patients from other regions and especially in developed countries [19–21, 30, 34]. This can be related to study population and periodontal therapy used. Some of the previous studies included smokers even if it is well known that nicotin contained in cigarette is responsible of delayed periodontal healing [35, 36]. On the other hand, patients treated by insulin were excluded. If periodontal treatment can improve insulin sensibility as showed by Iwamoto et al. [37], this can increase the effect of insulin therapy and thereby rapidly decreasing HbA1c levels [24].

One of the advantages of our study was the use of a subgingival irrigation which improves periodontal health in a better way when it is adjunct to periodontal treatment compared to periodontal treatment alone [38]. The effects of irrigation are more important if the irrigant is an antiseptic solution like the 10% povidone iodine solution used in this study [39]. Povidone iodine is a highly efficient microbicide by contact for gram-positive and gram-negative bacteria, fungi, mycobacteria, viruses and protozoans. Because of rapid microbial killing, a short term application of this agent alone may produce an adequate antimicrobial effect. In addition, povidone iodine is exempt of blood or saliva proteins deactivation and emergence of povidone iodine resistance micro-organism has not been reported to have been detected to date.

Since a significant drop in glycated haemoglobin of about 1 point is associated with a decrease of the risk of complications by 35%, a 25% reduction in diabetes-related deaths and a 18% reduction in combined fatal and non-fatal myocardial infarction [40], we can further evaluate and appreciate the benefit presented by the treatment of periodontitis in diabetic patients in our context [41]. This non-surgical periodontal treatment helps to reduce glycated haemoglobin by more than 2 points. Its use can therefore reduce cardiovascular and renal mortality related to diabetes as suggested by Saremi et al. in preceding decade [42]. This highlights the urgency of a greater sensitization on the importance of the treatment of chronic periodontitis in patients with diabetes and the necessity for physicians and other hospital practitioners to be awareness of this relationship and to integrate periodontal consultations in the monitoring and the overall management of the diabetic patient. It is imperative for physicians to consider periodontal structures as potential infection site as well as skin, urinary tract and lungs and to systematically check the presence of a periodontal infection.

In addition to the clinical benefit such practice also offers the advantage of a reduction of the cost of treatment for allowing a reduction of more than 1% of glycated hemoglobin, it is better than all oral anti-diabetic treatment currently available on the market of low developing country. Non-surgical treatment of chronic periodontitis in diabetic patients would therefore reduce the cost of treatment and would be a good alternative before applying second-line combination therapy for poorly controlled patients previously on monotherapy. Moreover, with no side effects, it has the advantage of a good tolerance and greater safety than oral anti diabetic drugs which are well known to have at least a notorious side effect.

Diabetes and periodontitis have some risk factors in common like obesity. In our population the mean of BMI was about 28.2 ± 5.4 kg/m^2^, obesity was found in 43.3% of the participants. It appears that those 3 states are linked by inflammation as demonstrated by Genco et al. [13] in their proposed model linking inflammation to obesity diabetes and periodontal infections. In this model, adipocytes secrete proinflammatory cytokines like TNF which is able to inhibit insulin signaling, leading to insulin resistance [13]. As time goes on, diabetes mellitus breaks out with another hyperinflammatory state and can be responsible of periodontitis infections onset. At the sight of these statements, we can think that the management of overweight or obesity can improve glycemic control in diabetic patients. More over the first lines for diabetic cares are non-pharmaceutical measures which include the diet. So, in this survey, the reduction of BMI could have contribute to glycemic control. However no significant drop in BMI values has been recorded after 3 months follow-up.

The patients included in this study had moderate to severe periodontitis according to CDC/AAP classification. However, as this case definition does not incorporate measurements from all the six sites, disease can be underestimate. In the CDC/AAP case definitions, the measurements of mid-bucal and mid –lingual are not includes, it means that furcation involvement is not assessed. Additionally, this case definition for periodontal status does not assess bleeding on probing so that informations about current inflammatory status are not incorporate. It means that this classification could not be suitable for a clinical trial but as we used complementary index like Ainamo and Bay index and evaluated changes in PPD and CAL means, the issues of our investigations are as attractive as expected.

As this study was design to evaluate the effects of periodontal treatment on glycated hemoglobin, we did not investigate the evolution of inflammation markers. Moreover, long term effects of the treatment were not determined. Meaning that the adoption of systematic screening and treatment or recommendations requires further studies at large-scale, randomized clinical trials, including measurements of inflammatory mediators to determine the relation between the reduction of HbA1c levels following periodontal therapy and inflammatory markers and longitudinal studies to determine long term effects of scaling and root planning associated to povidone iodine subgingival irrigation on glycaemic control.

## Conclusion

The results of this single-blinded randomized and controlled clinical trial show that the addition of periodontal therapy including subgingival irrigation with a 10% povidone-iodine solution to current medical therapy improves glycaemic control in sub-Saharan African type 2 diabetes patients with poorly controlled diabetes.

## Abbreviations

BMI: Body mass index
CAL: Clinical attachment loss
CDC-AAP: Center for Diseases Control- American academy of periodontology
CEJ: Cemento-enamel junction
GBI: Gingival bleeding index
GM: Gingival margin
HbA1c: Glycated haemoglobin
IL-1: Interleukin
IL-6: Interleukin 6
NSPT: Non-surgical periodontitis treatment
PD: Probing pocket depth
SD: Standard deviation
T2D: Type 2 diabetes
TNFα: Tumour necrosis factor-α
